# The complete mitochondrial genome of *Xyrias revulsus*（Jordan & Snyder, 1901）

**DOI:** 10.1080/23802359.2020.1806130

**Published:** 2020-08-12

**Authors:** Fangmei Liang, Lili Liu, Yanmei Wang, Pengliang Wang, Peng Zhu, Hong Zhang, Haiping Wu, Min Lu, Hongji Meng, Haihua Huang, Qiuhui Li, Youhou Xu

**Affiliations:** Guangxi Key Laboratory of Beibu Gulf Marine Biodiversity Conservation, Beibu Gulf University, Qinzhou, China

**Keywords:** *Xyrias revulsus*, mitochondrial genome, Illumina sequencing

## Abstract

*Xyrias revulsus* is one of the species in the China-Vietnam Collective Fishery Zone, which has a few relevant studies. In this study, the mitochondrial genome of *X. revulsus* was determined for the first time using next-generation sequencing; the overall base components of mitogenome consisting of 17,784 bp was 32.45% for A, 25.76% for T, 15.72% for G, 26.08% for C, and its GC content was 41.8%. The mitochondrial circular genome was composed of 13 protein-coding genes, 22 transfer RNAs, 2D-loop, and 2 ribosomal RNAs. Polygenetic analysis showed that the *X. revulsus* was very close to *Ophisurus macrorhynchos*. It can provide data reference for the analysis of genetic evolution of this species.

*Xyrias revulsus* is a member of Guilliformes, Ophichthidae, Xyrias, mainly distributed in the indo-pacific seas (Chen 2006). It is a rare species that inhabits waters from a few meters to 300 m depth (McCosker 1998). Little is known about it, which was first discovered in 1901 (Jordan and Snyder 1901). So far it is only reported in the east of Evans Shoal, the north of Bathhurst Island, the Izu Peninsula, Diaoyu Islands and the east China Sea (Huang and Zhan 1981; Tan 1983; Anonymous 1996; Mccosker et al. 2009). Little research has been done on the genome, population genetics and evolutionary relationships. Mitochondrial genomes has the advantages of stable structure and compact gene arrangement, which has been widely used in the study of Metazoan population genetics, biogeography and system evolution (Boore and Brown 1998; Curole and Kocher 1999; Boore et al 2005; Shen et al 2009). Hence, the complete mitochondrial genome sequence of *X. revulsus* was sequenced, assembled and characterized, which could provide important genetic data for studying of population genetics, molecular phylogenetics and evolutionary relationship of *X. revulsus*. All these provide important basic information for the conservation and utilization of this biome resource.

Total genomic DNA was isolated from each species using approximately of muscle tissue. Total DNA was eluted in sterile deionized water and was stored at −20 °C. The specimen was collected at the China-Vietnam Collective Fishery Zone (107.634 N, 19.714E) and stored at the Herbarium of Ocean College in Beibu Gulf University (X.R.001). Paired-end library (450 bp) was sequenced using Illumina Hiseq4000 platform, with 150 bp pair-end sequencing method. The mitochondrial genome was assembled using the chloroplast and mitochondrion assemble (CMA)V1.1.1 software (Guangzhou SCGene Co., Ltd), which was based on sequencing reads’ overlap and paired-end relationship. Protein-coding genes and rRNA genes were annotated with blast+(2.5.0) with allied species, and tRNAs were predicted with tRNAscan-SE v2.0 (http://lowelab.ucsc.edu/tRNAscan-SE/) (Lowe and Chan 2016). 11,226 raw reads with an average length of 150 bases and 1,683,900 nt were obtained, with average reads depth of 94.7X. Our research findings revealed that the circular genome is 17,784 bp, which consists of 13 protein-coding genes(PCGs), 2 D-loop, 2 rRNAs genes, 22 tRNAs genes, showing that the gene composition and arrangement are very close to the reported *Ophisurus macrorhynchos* composition (Shen et al. 2014). The gene composition of eels mitochondrial genomes is conservative．The contents of A, T, G, and C in mitochondrial genome were 32.45%, 25.76%, 15.72%, and 26.08%, respectively. An overall GC content of whole mitochondrial genome is 41.8%. The sequence was deposited in the GenBank (GenBank: KY021065). A phylogenetic analysis was conducted on 20 mitochondrial genomes. Maximum likelihood (ML) method was used for phylogenetic analysis. The best-fit models of evolution for the coding genes was selected by jmodeltest2 (https://github.com/ddarriba/jmodeltest2) (Darriba et al. 2012). RAxML v8.0.0 (RAxML Version 8 2014) was used to build the tree with 1000 bootstrap (Figure 1). Polygenetic analysis showed that the *X. revulsus* was very close to *O. macrorhynchos*. The complete mitochondrial genome of *X. revulsus* provided important genetic information for understanding phylogenetic relationships of Prosobranchia mitochondrial genome.

**Figure 1.**
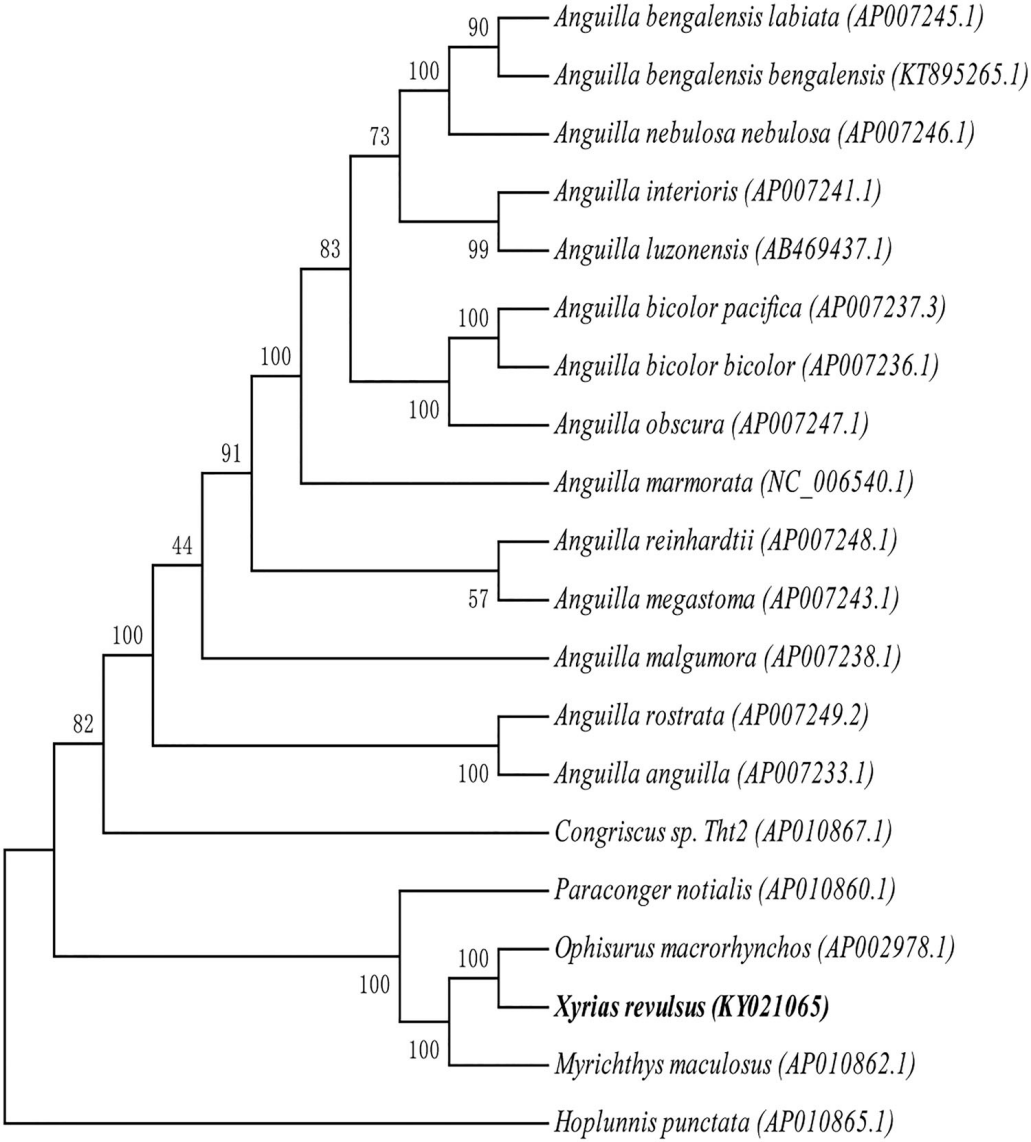
Phylogenetic relationships of Prosobranchia based on 20 mitochondrial genomes using NJ method. GenBank accession numbers: *Anguilla bengalensis labiata* (AP007245.1), *Anguilla bengalensis bengalensis* (KT895265.1), *Anguilla nebulosa nebulosa* (AP007246.1), *Anguilla interioris* (AP007241.1), *Anguilla luzonensis* (AB469437.1), *Anguilla bicolor pacifica* (AP007237.3), *Anguilla bicolor bicolor* (AP007236.1), *Anguilla obscura* (AP007247.1), *Anguilla marmorata* (NC_006540.1), *Anguilla reinhardtii* (AP007248.1), *Anguilla megastoma* (AP007243.1), *Anguilla malgumora* (AP007238.1), *Anguilla rostrata* (AP007249.2), *Anguilla anguilla* (AP007233.1), *Congriscus sp. Tht2* (AP010867.1), *Paraconger notialis* (AP010860.1), *Ophisurus macrorhynchos* (AP002978.1), *Xyrias revulsus* (KY021065.1), *Myrichthys maculosus* (AP010862.1), *Hoplunnis punctate* (AP010865.1).

## Data Availability

Our data is shared in ethically correct circumstances, without violating human protection, or other valid ethical, privacy, or security issues. The data that support the findings of this study are openly available in NCBI (https://www.ncbi.nlm.nih.gov/nuccore/KY021065.1/).
